# Circulating miR-146b and miR-27b are efficient biomarkers for early diagnosis of Equidae osteoarthritis

**DOI:** 10.1038/s41598-023-35207-3

**Published:** 2023-05-17

**Authors:** Aya M. Yassin, Huda O. AbuBakr, Ahmed I. Abdelgalil, Omar A. Farid, Adel M. El-Behairy, Eman M. Gouda

**Affiliations:** 1 Department of Biochemistry and Molecular Biology, Faculty of Veterinary Medicine, Cairo University, Giza, 12211 Egypt; 2 Department of Surgery, Anesthesiology, and Radiology, Faculty of Veterinary Medicine, Cairo University, Giza, 12211 Egypt; 3 Department of Physiology, National Organization for Drug Control and Research, Giza, Egypt

**Keywords:** Biochemistry, Genetics, Molecular medicine, Rheumatology

## Abstract

One of the most orthopedic problems seen in the equine is osteoarthritis (OA). The present study tracks some biochemical, epigenetic, and transcriptomic factors along different stages of monoiodoacetate (MIA) induced OA in donkeys in serum and synovial fluid. The aim of the study was the detection of sensitive noninvasive early biomarkers. OA was induced by a single intra-articular injection of 25 mg of MIA into the left radiocarpal joint of nine donkeys. Serum and synovial samples were taken at zero-day and different intervals for assessment of total GAGs and CS levels as well as miR-146b, miR-27b, TRAF-6, and COL10A1 gene expression. The results showed that the total GAGs and CS levels increased in different stages of OA. The level of expression of both miR-146b and miR-27b were upregulated as OA progressed and then downregulated at late stages. TRAF-6 gene was upregulated at the late stage while synovial fluid COL10A1 was over-expressed at the early stage of OA and then decreased at the late stages (P < 0.05). In conclusion, both miR-146b and miR-27b together with COL10A1 could be used as promising noninvasive biomarkers for the very early diagnosis of OA.

## Introduction

Osteoarthritis (OA), as one of the most heterogeneous and prevalent degenerative joint diseases, is of major concern for human health. It is also one of the most common orthopedic problems seen in horses^1^. Clinical signs of disease are lameness, joint swelling, pain on flexion, or reduced activity, and with time, these symptoms lead to structural joint alterations. Degradation of articular cartilage is a consequence of its poor capacity to repair and withstand the cyclic trauma of athletic activity, and this is exacerbated with aging^2^. In the clinical routine, no available sensitive and specific blood biomarkers can detect the disease's initial stages or predict the future development of OA. Consequently, there is great interest in the identification of new markers^3^.

Genetic variation studies have identified many candidate genes in defined radiographic cases with small effects on the overall risk of OA. However, these genes cannot fully and clearly explain the strong genetic component that is implicated in OA onset and progression^4^. Therefore, other mechanisms have been involved, such as epigenetics. Epigenetics refers to the covalent modification of DNA, proteins, or RNA without altering their primary sequences, resulting in changes to the function and/or regulation of these molecules. The most studied epigenetic factors are DNA methylation and non-coding RNAs^4,5^.

Chondrocytes in cartilage normally function to produce and secrete extracellular matrix proteins to maintain cartilage integrity^6^. During the disease conditions, the chondrocytes change their behavior; they become autophagic, overexpress the hypertrophy markers as COLX, and secrete the inflammatory cytokines or small fragments of nucleic acid as microRNAs (miRNAs) to promote cell degeneration and apoptosis^7,8^.

MicroRNAs (miRs) are small non-coding RNAs of approximately 22 nucleotides in length that can silence gene expression by binding to a complementary sequence in the 3′ untranslated regions (3′-UTR) of target mRNAs, resulting in translational repression or target degradation^9,10^. miRNAs can be found intracellularly or extracellularly, circulating in virtually any biological fluid in a remarkably stable manner^11^. Many reviews mentioned the involvement of miRNAs in normal conditions of the articular cartilage (osteoblastogenesis, osteoclastogenesis, and chondrogenesis) as well as in pathological conditions (cartilage degradation, synovial inflammation, and OA progression)^12–14^. Many miRNAs, including miR-101^15^, miR-181c^16^, miR-675^17^, miR-770^18^, miR-140^19^, miR-146a^20^, miR-27a^21^, miR-122^22^, miR 130a^23^, miR-15a^24^, and miR-127-5P^25^ have been reported to be engaged in OA pathogenesis through regulation of cartilage homeostasis, chondrocyte metabolism, and inflammatory responses as well as proteolytic enzyme activity.

Because biological fluids are generally obtainable through minimally invasive techniques, circulating miRNAs are attractive candidates for disease diagnosis, monitoring, and prognostication^26,27^. Several studies have investigated circulating miRNAs, Murata et al. reported that differentially expressed miRNAs in the synovial fluid and the plasma could distinguish individuals with OA or RA from healthy controls^28^. Similarly, Li et al. identified OA-specific miRNAs in synovial fluid that are differentially regulated in early and late-phase OA^29^. Ntoumou et al. found 279 miRNAs differentially expressed in the serum of osteoarthritic conditions, and 3 signature miRNAs (140-3p, 33b-3p, and 671-3p) were down-regulated. These miRNAs are known to be involved in several molecular pathways, including Wnt, ErbB, and TGF-beta^30^.

Synovial fluid miRNAs such as miR-29b3p and miR-140 were suggested as OA biomarkers and strongly correlated with radiographic knee OA severity^31,32^. Equine miRNAs have been studied in healthy tissues^33,34^ and in different diseases, including osteochondrosis, rhabdomyolysis, and insulin resistance^35–37^. Castanheira et al. revealed 22 differentially expressed sncRNAs in equine synovial fluid as well as validated that miR-223 was significantly reduced in early osteoarthritis and miR-23b, let-7a-2, snord96A, and snord13 were significantly upregulated^38^. MiR-146b has been reported to play a role in the regulation of cell differentiation, proliferation, and migration in various tumor cells^39,40^. MiR-146b has also been identified as being involved in the chondrogenic differentiation of human bone marrow-derived SSCs through the modulation of SOX5^41^.

MiR-27b, a chondroprotective miRNA, targets MMP13 and is negatively correlated with its expression. The activated NF-κB could up-regulate the expression of MMP-13 but act as a negative regulator of miR-27b in promoting chondrocyte degradation^42^.

One of the main hindering issues in the development of efficient therapeutic regimes for OA is the inability to detect and track the early, pre-clinical changes of the disease^43^. Profiling the synovial fluid within the affected joint at the early stages of the disease may provide new insights into the pathological changes occurring during OA initiation and progression and help develop new therapeutic approaches; however, it is the most difficult to assess because of the invasive techniques involved with its collection. Meanwhile, the serum is easily accessible because it can be extracted from the body with minimally invasive techniques and is also the site of much of the body’s metabolism; therefore, many of the changes that might occur with OA can be represented by serum biomarkers. Based on our previous work^1^, the current study aims to track the biochemical, epigenetic, and transcriptomic changes along the different stages of OA in serum and synovial fluid in MIA-induced OA donkeys for the establishment of noninvasive biomarkers for early diagnosis and prognosis of OA.

## Methods

### Ethical statement

The present study was approved by the Institutional Animal Care and Use Committee of Cairo University (CU-IACUC) and was performed after receiving ethical approval (approval number: CU/II/F/4/16). The experiment was performed following ARRIVE’s relevant guidelines and regulations.

### Experimental study design

Based on our previous study and experimental design of equine osteoarthritis that was recently published by Yassin et al.^1^, The current study was conducted for the investigation of some biochemical, transcriptomic, and epigenetic biomarkers in synovial fluid and serum samples. Briefly, nine healthy adult male local breed donkeys, aged 3–5 years and weighing 150–200 kg, were subjected to the study following the clinical assessment. Osteoarthritis was chemically induced through intraarticular injection of a single dose of MIA (25 mg/ml) (sodium monoiodoacetate, Sigma-Aldrich, St. Louis, MO, USA) at the left radiocarpal joint. Synovial fluid samples were collected from the left radiocarpal joint, and blood samples were collected from the left jugular vein on plain tubes and then centrifuged at 1500×*g* for 15 min. Samples were collected at day 0 (before the OA induction), then in the 1st week, 1st, 2nd, 3rd, 5th, and 7th months after the induction. Both samples were kept at − 80 °C for further analysis.

### Biochemical analysis

The concentration of total glycosaminoglycans (GAGs) and chondroitin sulfate (CS) was assessed by High-performance liquid chromatography (HPLC) on Day 0, 1st week then at the 1st, 2nd, 3rd, 5th, and 7th months post-MIA-injection as modified according to Gässler et al.^44^. Briefly; serum and synovial samples were dissolved with 1:1 a ratio for the sample to solvent (deionized water: acetonitrile 80:20%) and then centrifuged at 1957×*g* for 10 min. The clear supernatants were ready for analysis by the isocratic HPLC system (Agilent 1200 Series equipped with the computerized solvent delivery system and UV detector, Santa Clara, CA, USA) using Supelco Kromasil NH2 silica gel column (5 µm particle size, pore size 100 A) with flow rate 1.5 ml/min. The effluent is monitored by a UV detector at wavelength 195 nm by using GAGs and CS standard solutions (Sigma Aldrich, St. Louis, MO, USA) and the injection volume was 20 µl.

### Transcriptomic and epigenetic quantitative real-time polymerase chain reaction (qRT-PCR) of miRNAs and their target genes

Total RNA and miRNAs in serum and synovial fluid samples were extracted using miRNeasy Mini Kit (Qiagen, Hilden, Germany cat. no. 217004) according to the manufacturer’s protocol with Serum/Plasma Spike-In Control for data normalization (Qiagen, cat. no. 219610). Total RNA purity and concentration were obtained using a nanodrop ND-1000 spectrophotometer. The isolated RNA was used for cDNA synthesis of target genes using Revert Aid Reverse Transcriptase (Thermo Scientific, Cat. No. EP0441, USA) according to the provided guidelines. Real-time PCR (qPCR) was performed in a total volume of 20-μl using a mixture of 1 μl cDNA, 0.5 mM of each primer (Table 1), iQ SYBR Green Premix (Thermo Scientific, Cat. No. K0221, USA).

**Table 1:** Primer sequences of reference, COL10A1, and TRAF-6 genes of Equus asinus.

Target genes	Accession no.	Sequence (5′ to 3′)	Product size (bp)
β-actin (ACTB) as (reference gene)	XM_014835097.1	F: 5′CGACATCCGTAAGGACCTGT3′ R: 5′GTGGACAATGAGGCCAGAAT3′	192
COL10A1	XM_014866148.1	F: 5′GGAGAAAGGGGTTTCTCTGG3′ R: 5′ACCATTGTTTCCAGGCACTC3′	183
TRAF-6	XM_014851023.1	F: 5′ ATCCCACGGAACCCAAAA 3′ R: 5′ CCCCAGTATCAGTGCTTCGT 3′	175

TaqMan^®^ microRNA Reverse Transcription kit was used for miRNA reverse transcription of the isolated RNA using (Applied Biosystems, cat. no.4366596) as manufacture protocol. The sequence-specific RT primer was provided in each TaqMan^®^ microRNA ready-made assay (sequence-specific primers/probes); hsa-miR-146b (assay ID: 001097) and hsa-miR-27b (assay ID:000409). Accurate and reliable results were obtained through the normalization of RT-qPCR data with spike-in control cel-miR-39 (assay ID:000200). Taqman Real-time PCR (qPCR) was performed in a total volume of 20 μl using a mixture of 1.33 μl cDNA,10 μl TaqMan^®^ universal PCR Master Mix II, No UNG (2X) (Applied Biosystems, Belgium, cat. no. 4440043), 1 μl TaqMan MicroRNA Assay Mix.). PCR amplification and analysis of total and miRNAs expression were achieved using Bio-Rad iCycler thermal cycler and the MyiQ real-time PCR detection system. Each assay includes triplicate samples for each tested cDNAs and no-template negative control, the expression relative to the control is calculated using the equation 2^−ΔΔCT^^45^.

### Statistical analysis

The obtained data were statistically analyzed using the One-Way ANOVA Statistics, version 24.0 software (SPSS Inc., Chicago, IL, USA). Polynomial contrasts, post-hoc Duncan, and descriptive statistics were performed. The level of significance was set at p ≤ 0.05. The represented values are given as a standard error of the mean (SEM).

## Results

### Biochemical findings of total GAGS and CS concentrations in serum and synovial fluid

**Figure 1:**
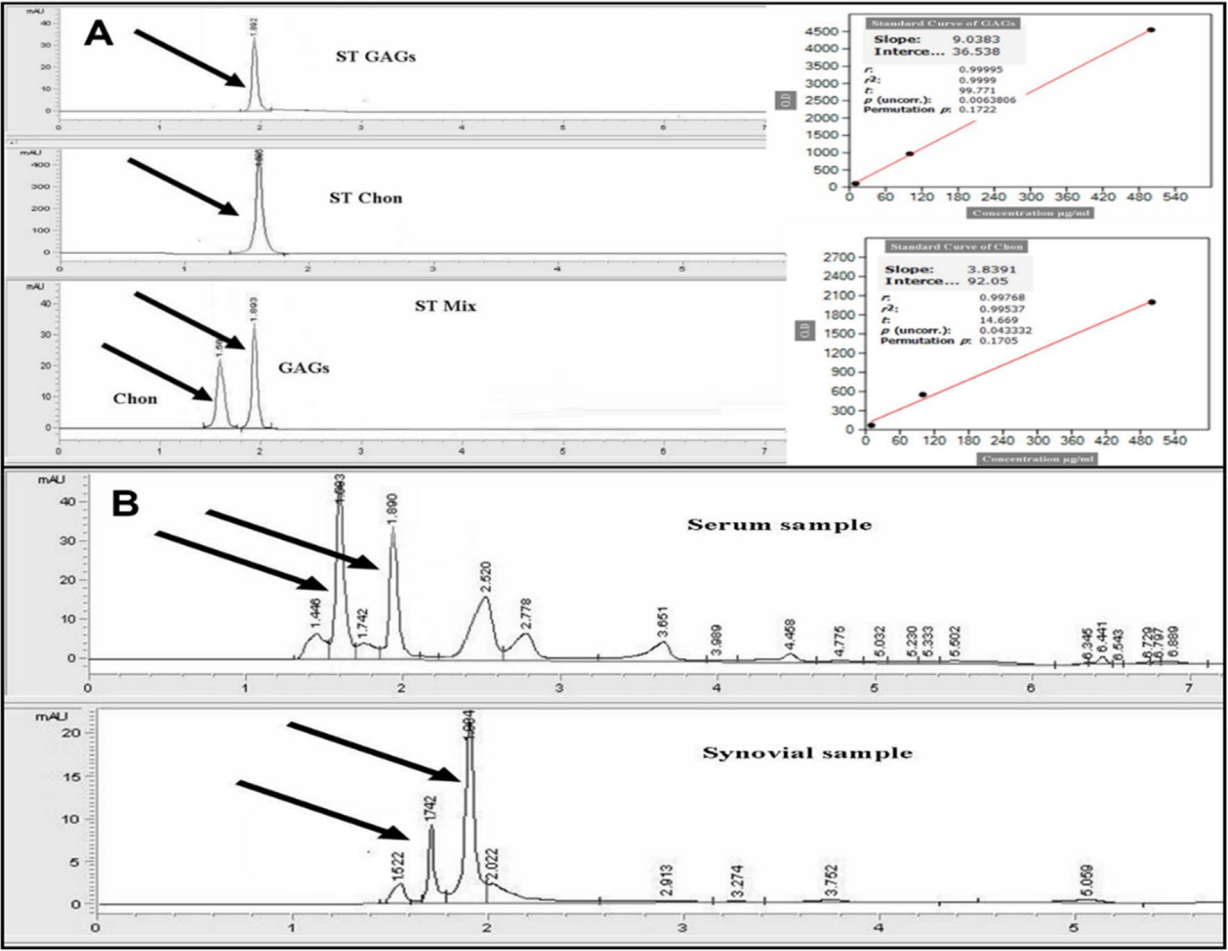
(**A**) Chromatograms for total GAGs and Chondroitin Sulphate standards. (**B**) chromatograms for total GAGs and chondroitin sulphate in serum and synovial fluid samples.

**Figure 2:**
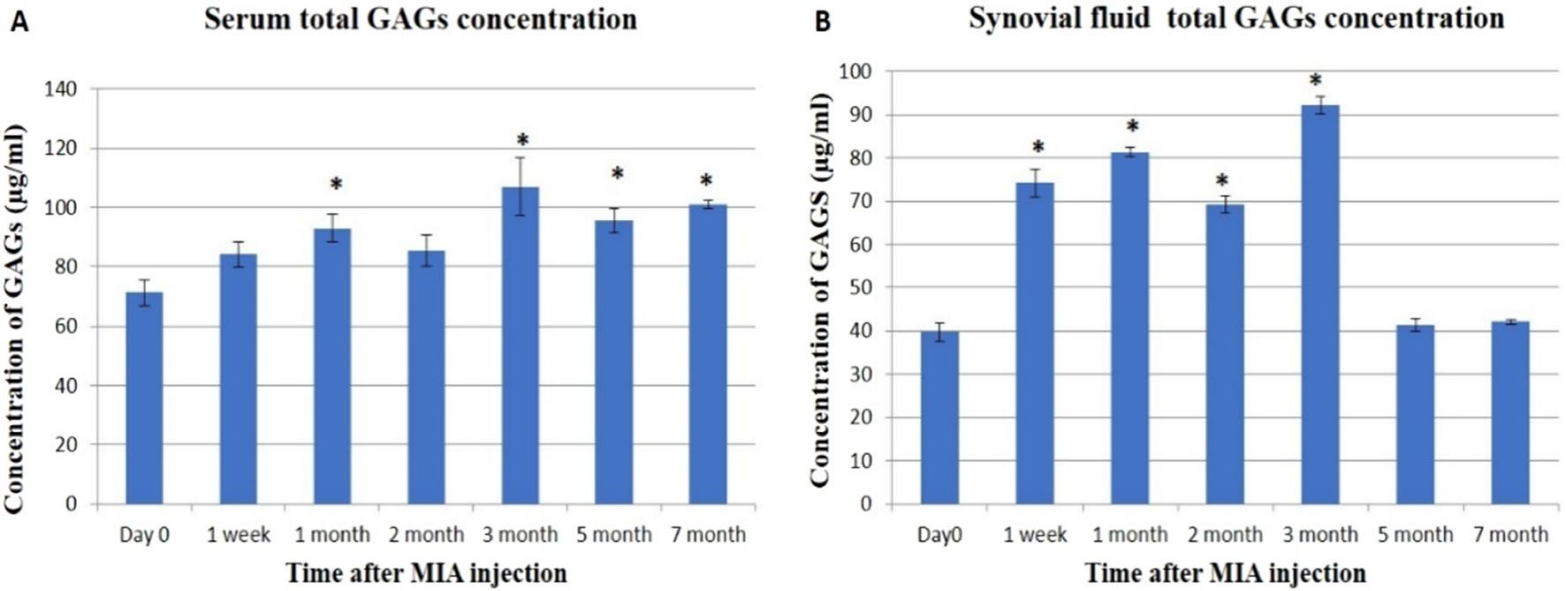
The concentration of total GAGs (µg/ml) by HPLC. (**A**) The concentrations of total GAGs in serum samples. (**B**) The concentrations of total GAGs in synovial fluid samples. Data are represented as mean value ± standard error (S.E.) where (n = 3). (*) denotes a significant difference from control samples at day 0 at p < 0.05.

In serum and synovial fluid samples; the concentration of total GAGs recorded the highest significant concentration at the 3rd month post-MIA injection (Figs. 1, 2).

**Figure 3:**
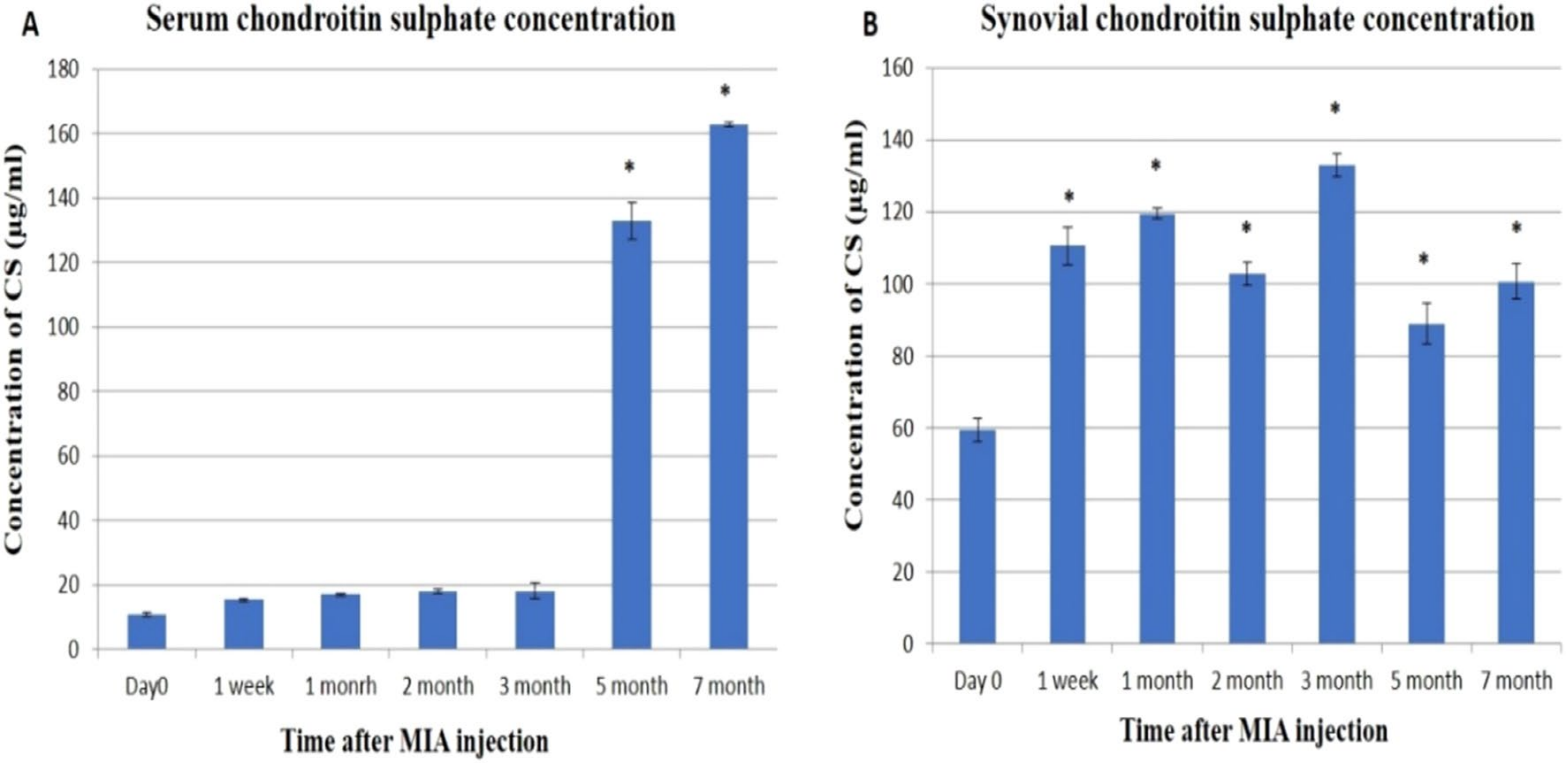
The concentration of chondroitin sulphate (µg/ml) by HPLC. (**A**) The concentration of chondroitin sulphate in serum samples. (**B**) The concentration of chondroitin sulphate in synovial fluid samples. Data represented as mean value ± standard error (S.E.) where (n = 3). (*) denotes a significant difference from control samples at day 0 at p < 0.05.

The concentration of CS in serum was significantly increased only at the 5th and 7th month (Fig. 3A), while in the synovial fluid; CS showed an irregular significant increase from day 0 with the highest significant level at the 3rd month (Fig. 3B).

### Transcriptomic and epigenetic qRT-PCR of miRNAs and their target genes findings

#### Expression levels of miR-146b and miR-27b

**Figure 4:**
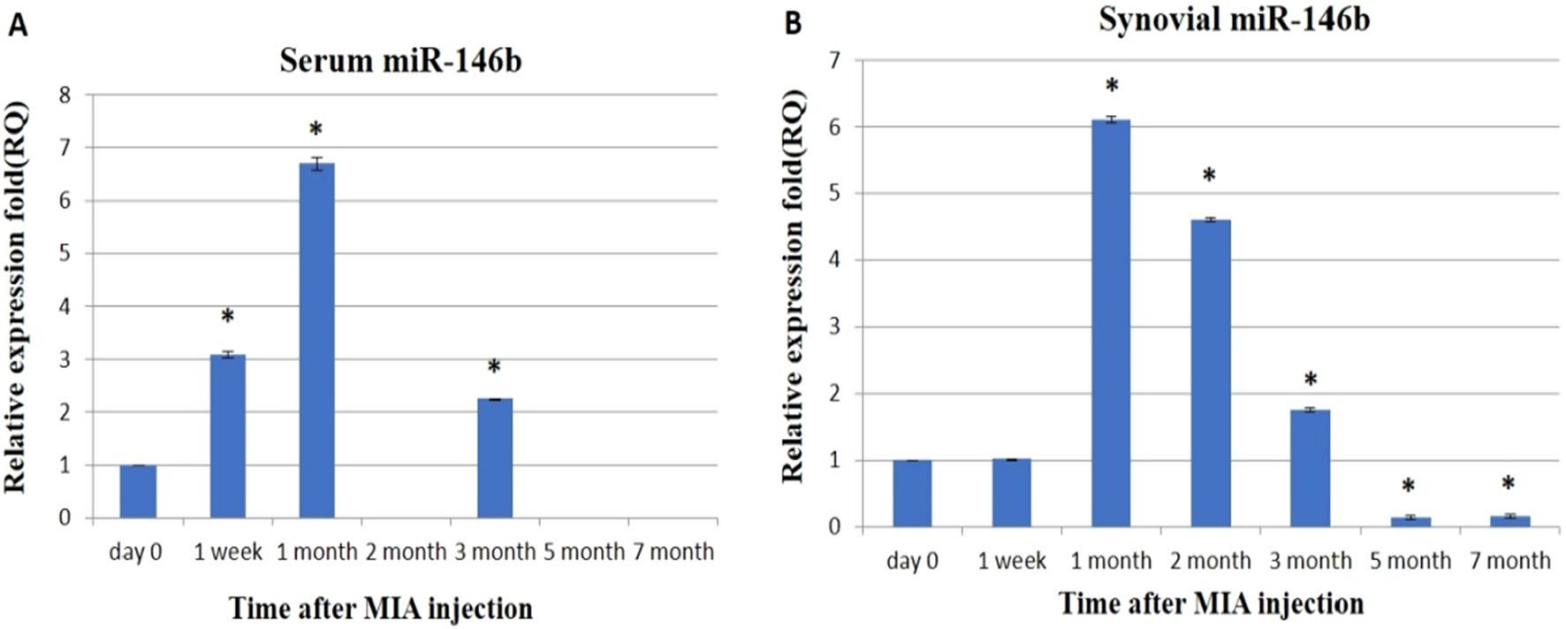
The effect of MIA on the relative expression level of miR-146b gene: qRT-PCR results for miR-146b gene before MIA injection (Day 0) and post-MIA injection at 1st week, 1st, 2nd, 3rd, 5th, and 7th months in serum (**A**) and synovial fluid (**B**). Data represented as mean value ± standard error (S.E.) where (n = 3) of triplicate experiments. (*) denotes a significant difference from control samples at day 0 at p < 0.05.

In serum, the expression level of miR-146b was significantly up-regulated immediately at 1 week,1 month, and 3 months after MIA injection by 3.1-, 6.7-, and 2.26-folds, respectively. While at the 2nd, 5th, and 7th months; the expression level of miR-146b was not detected (Fig. 4A). In the synovial fluid sample, miR-146b was recorded to be significantly up-regulated starting from the first month till the 3rd month by 6.1-, 4.6-, and 1.76-folds respectively. While at the 5th and 7th months, the expression of miR-146b was significantly down-regulated to 0.13- and 0.16-folds, respectively (Fig. 4B). Herein, the maximum significant expression of serum and synovial fluid samples for miR-146 was observed in the 1st month.

**Figure 5:**
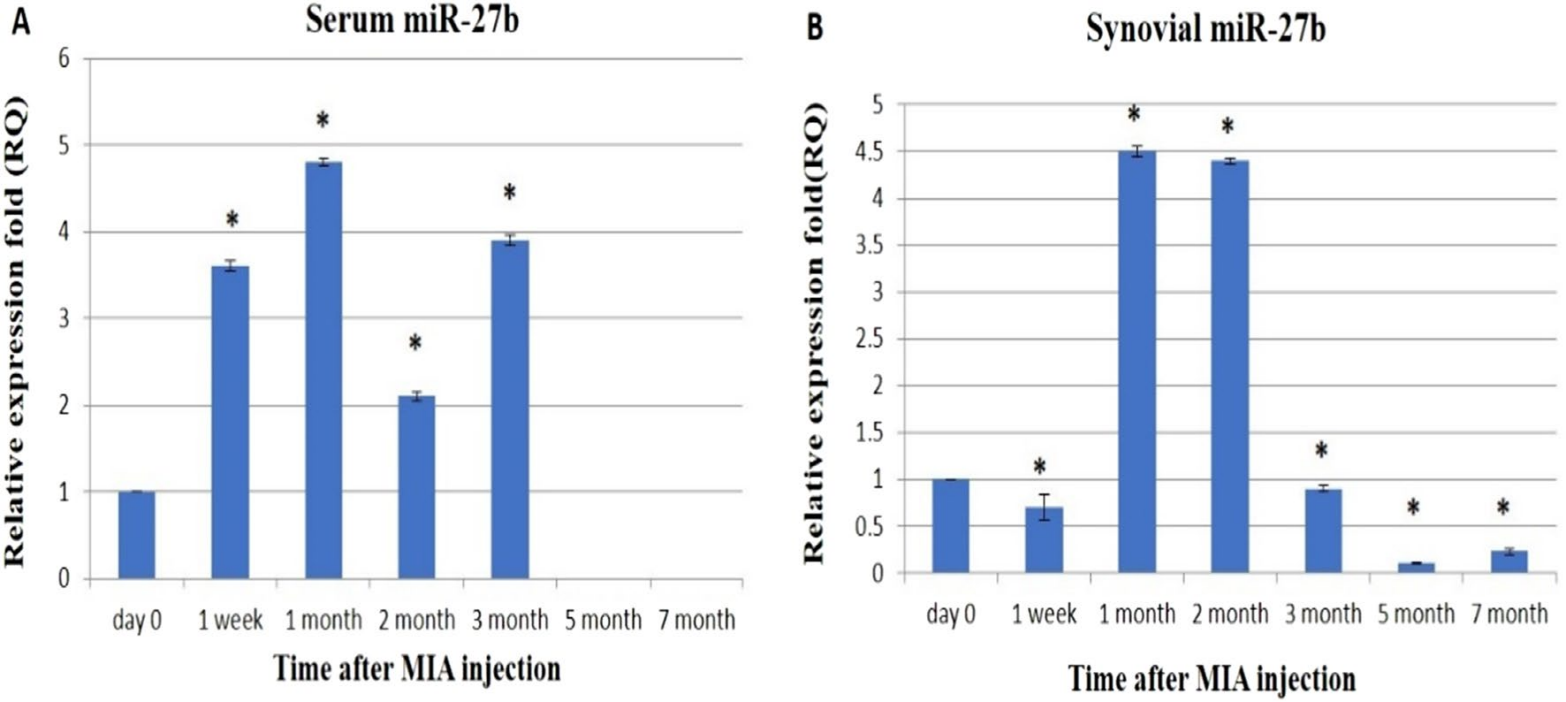
The effect of MIA on the relative expression level of miR-27b gene:qRT-PCR results for miR-27b before MIA injection (Day 0) and post-MIA injection at 1st week, 1st, 2nd, 3rd, 5th, and 7th months in serum (**A**) and in synovial fluid (**B**). Data represented as mean value ± standard error (S.E.) where (n = 3) of triplicate experiments. (*) denotes a significant difference from control samples at day 0 at p < 0.05.

The expression level of miR-27b in serum and synovial fluid samples was investigated throughout the experiments. The results from serum samples showed that miR-27b was significantly up-regulated rapidly 1-week post-MIA injection up to the third month by 3.6-, 4.8-, 2.1-, and 3.9-folds, respectively. Moreover, our data revealed that miR-27b expression was not detected in the 5th and 7th months (Fig. 5A). While in the synovial fluid samples, the miR-27b up-regulation was identified significantly only on the 1st and the 2nd-month post-injection by 4.5- and 4.4-fold, respectively. The 1st week and the 3rd, 5th, and 7th months exhibited significant down-regulation to 0.7-, 0.9-, 0.11-, and 0.23-folds, respectively (Fig. 5B). For serum and synovial fluid samples, the maximum significant up-regulation for miR-27b was observed in the 1st month.

#### The expression level of TRAF-6 and COL10A1 genes

**Figure 6:**
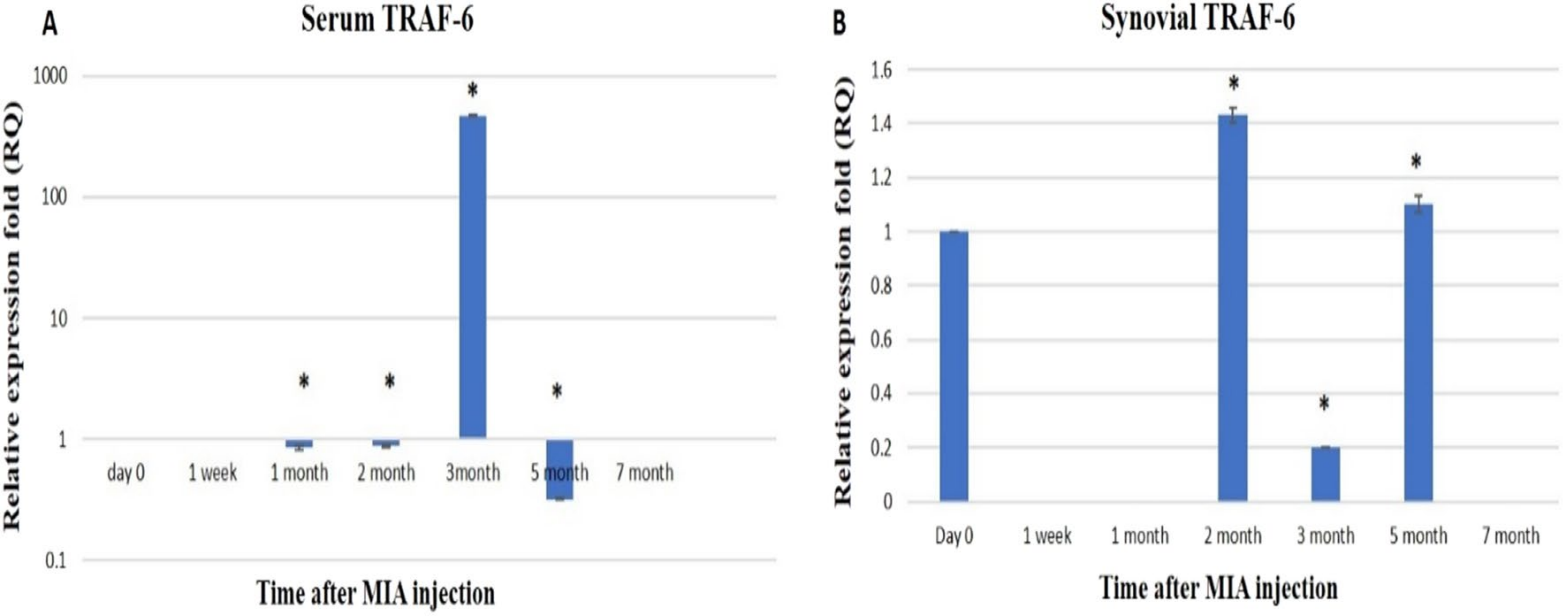
Graphical representation for the effect of MIA on the relative expression level of the TRAF-6 gene. qRT-PCR results before MIA injection (Day 0) and post-MIA injection at 1st week,1st, 2nd,3rd, 5th, and 7th months in serum (**A**) and synovial fluid (**B**). Data represented as mean value ± standard error (S.E.) where (n = 3) of triplicate experiments. (*) denotes a significant difference from control samples at day 0 at p < 0.05.

Our study showed that the expression of serum TRAF-6 was significantly up-regulated by 471.8-fold in the third month while significant down-regulation in 1st month,2nd month, and 5th month by 0.853-, 0.88-, and 0.32-folds, respectively. Whereas, its expression was not detected in the 2nd and the 7th month (Fig. 6A). Regarding synovial TRAF-6 expression level, it was up-regulated significantly at the 2nd and 5thmonth post-MIA injection by 1.4-fold and 1.1-fold respectively, and down-regulated significantly at the 3rd month by 0.2-fold. While, the TRAF-6 expression was not detected in 1st week,1st month, and 7th month (Fig. 6B).

**Figure 7:**
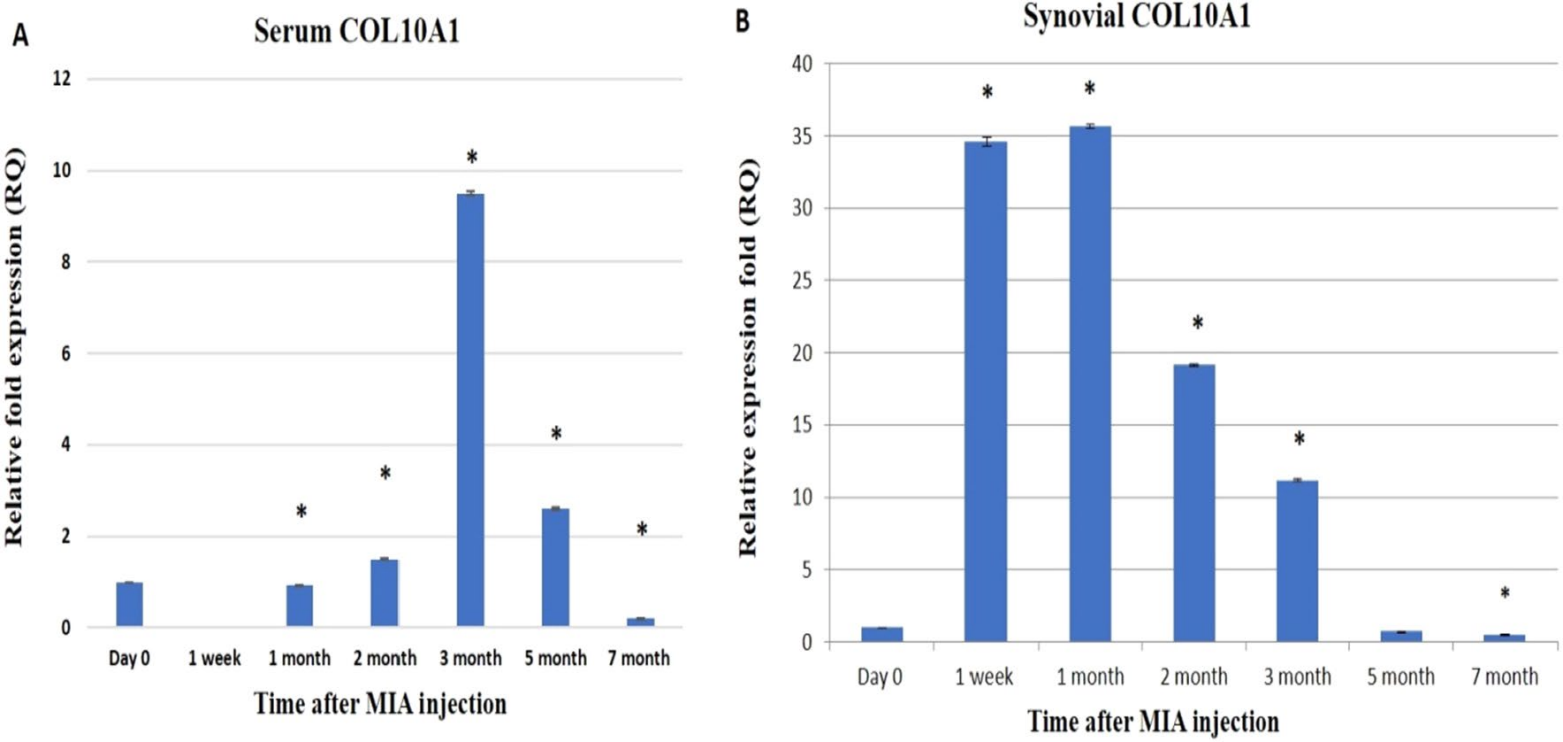
Graphical representation of the effect of MIA on the relative expression level of the COL10A1 gene in serum. qRT-PCR results before MIA injection (Day 0) and post-MIA injection at 1st week, 1st, 2nd, 3rd, 5th, and 7th months in serum (**A**), and synovial fluid (**B**). Data represented as mean value ± standard error (S.E.) where (n = 3) of triplicate experiments. (*) denotes a significant difference from control samples at day 0 at p < 0.05.

In serum samples, COL10A1 was observed to be significantly up-regulated at the 2nd, 3rd, and 5th months post-MIA injection by 1.5-, 9.5-, and 2.6-folds, respectively. Also, it was noticed that the periods of downregulation were at the 1st month and 7th month by 0.93- and 0.2-folds, respectively, and were not detected in the 1st week (Fig. 7A). In synovial fluid samples, COL10A1 was immediately up-regulated following MIA injection up to the 3rd month by 34.6, 35.6, and 19.14, respectively then it was down-regulated at the 5th and 7th month by 0.72- and 0.48-folds, respectively(Fig. 7B).

## Discussion

Osteoarthritis is characterized by the progressive degeneration of the articular cartilage matrix, resulting in the release of its components and degradation products, which can be measured biochemically to monitor disease progression^46^.

In the present study, serum and synovial fluid total GAGs in a model of MIA-induced OA in donkeys increased, especially by the 3rd month, then declined in the 5th and 7th months. High levels of GAGs were recorded in chronic and acute joint diseases^47^; in synovial fluid, serum, and urine of horses with OA^48,49^. While the decline in the synovial fluid total GAGs at the 5th and 7th months can be attributed to the ‘floor effect’ which is characterized by complete cartilage wear-out and loss of joint space as shown in the SF of patients with grade IV OA^50,51^. Also, these results came in context with our previous histopathological findings, which revealed the loss of proteoglycan from the articular cartilage^1^.

Concerning the findings of CS, serum CS was found to be significantly increased in the 5th and 7th months. In this context, serum CS was reported to be elevated in inflammatory diseases^28^ and all OA patients^52^. While our results concerning synovial CS showed that its levels tended to be elevated throughout the experimental period without any discrimination between the early and late osteoarthritic changes. This is consistent with a study on polo horses, which found an increase in synovial CS related to the inflammatory process of joint tissues^53^.

Several miRNAs have been involved in bone and cartilage homeostasis and development as well as the progression of OA through the modulation of osteoblastogenesis, osteoclastogenesis, chondrogenesis, synovial inflammation, and cartilage degradation^54^. Some miRNAs have been shown to exhibit anti-inflammatory effects in the synovium during OA. miR-146a/b is considered one of the key regulators in the NF-κB inflammatory response pathway by binding to the 3′ UTR of their mRNA targets, TRAF-6 and IRAK-1, resulting in their inhibition^55^.miR-146b shares sequence similarities with miR-146a, so miR-146b might be up-regulated in OA and target the pro-inflammatory mediators acting as an anti-inflammatory mediator^56^.

In the present study, miR-146b was upregulated in serum at the 1st week, 1st month, and 3rd month, and in synovial fluid at the 1st, 2nd, and 3rd month, while it wasn’t detected at the 2nd, 5th, and 7th months in both serum and synovial samples.

In accordance with our results, Overexpression of miR-146 at early OA was reported in human PBMCs (peripheral blood mononuclear cells)^57^, in the knee joint destabilization OA model^58^, in chondrocytes isolated from the human articular cartilage with OA^56^ and knocked-down in mouse models^59^. Because of their accumulation in the synovium in the early stages of OA, these miR-146 expressed in the circulating PBMCs of OA patients may contribute to the pathogenesis of OA^57^. Meanwhile, miR-146b down-regulation was observed during the chondrogenic differentiation of human bone marrow-derived SSCs^56^.

Regarding TRAF-6 expression, our findings demonstrated the strong correlation of miR-146b expression with the expression of the TRAF-6 gene after OA induction. The upregulation of miR-146b was coupled with the downregulation of TRAF-6 expression in the early stage of OA all over the 1st month in serum and synovial fluid samples. The significant up-regulation of TRAF-6 expression was in the 3rd month, the late stage of OA, for serum, and in the 2nd month, the early stage, for synovial fluid.

TRAF-6 is considered a critical intracellular mediator for the inflammatory pathways via playing the main role in the activation of NF-κB induced by lipopolysaccharide (LPS) /Toll-like receptor (TLR)-4 signaling and in the resorption activity of osteoclasts^60^. Herein, the inhibition of TRAF-6 brings antiapoptotic, anti-catabolic, and anti-inflammatory effects in the chondrocyte injury model by regulating the NF-kB signaling pathway^61^.

These results are in accordance with a previous study by Zhu et al.^62^ which demonstrated that TRAF-6 expression in RA synovium was significantly higher than that in OA synovium**.** Notably, overexpression of miR-146a in OA FLS decreased the expression of many inflammatory mediators, including TRAF-6, IRAK-1, COX-2, IL-8, MMP-13, and ADAMTS5 expression^63^. Besides, TRAF-6 was upregulated in osteoarthritis patients, which indicated that it may be associated with OA^64,65^ and FJOA^61^ severity and progression.

Many studies refer to miR-146a as a novel target in OA by negative feedback regulation of inflammatory responses^63,66–68^ or by promoting chondrocyte autophagy^69^. Other studies showed that miR-146 may contribute to OA pathogenesis by promoting the expression of VEGF and impairing the TGF-β signaling pathway via targeting Smad4^70,71^

In contrast to our findings, Liu et al*.* reported that miR-146b was up-regulated in IL-1β-treated chondrocytes and significantly affected cell viability and matrix gene expressions in chondrocytes. The downregulation of miR-146b profoundly inhibited caspase activation and the expression of the proteolytic enzymes. Moreover, intra-articular injection of antago-miR-146b protected mice with OA from cartilage degradation as well as proteoglycan loss, suggesting that up-regulation of miR-146b can contribute to the development and progression of OA^41^**.** Meanwhile in our previous investigation^1^; we found that MMPs activity and Caspase-3 expression increased, accompanied by articular cartilage degeneration and loss of proteoglycan at the 5th and 7th months where the miR-146b was downregulated in the present study.

The non-expected expression pattern of miR-146 and TRAF-6 at the late stage of OA can be explained by the fact that one miRNA could potentially control the expression of a few to several thousand genes. Conversely, each mRNA could be affected by multiple miRNAs^72^.

Few studies have investigated the expression level of miR-27b in OA chondrocytes and OA synovial fluid^29,42,73,74^. In the current study, serum, and synovial fluid miR-27b expression showed the maximum up-regulation at the 1st-month post-MIA induction of OA in donkeys and then declined. In context, miR-27b was up-regulated in the synovial fluid and down-regulated in the chondrocytes following IL-1β stimulation, which could be explained as miRNAs that respond to inflammatory stimuli (IL-1β) were released into the synovial fluid from the synovium during OA in attempts from the chondrocyte to compensate for the catabolic actions^29^.

Zhou et al. demonstrated that miR-27b directly inhibits chondrocyte apoptosis and, thus, ameliorates the development of RA^74^. Besides, the expression of miR-27b was sharply down-regulated, and the production of MMP-13 protein was enhanced in normal or OA chondrocytes stimulated with IL-1β^42^.

We previously found that MMP-13 was highly up-regulated in the 2nd month of MIA OA^1^, despite profound up-regulation for synovial miR-27b. These unexpected expression patterns of MMP-13 and miRNA-27b could be explained as synovial miR-27b did not correspond to the MMP-13 activity in the synovial fluid, but it was correlated to the miR-27b expressed in the chondrocytes and cannot be anticipated as a perfect one-to-one correspondence^29^**.** In addition, miR-27b interacts with the 3-UTR of MMP-13 mRNA with imperfect complementation and down-regulates its expression at the post-transcriptional level^42^. The context scores of miR-27b recorded low values among the microRNAs predicted to target the conserved sites in the 3-UTR of MMP-13 mRNA^42^, giving the chance for other miRNAs (miR-181b)^75^ and miR-33a^76^ to directly or miR-145^77^ indirectly increase MMP-13 expression. Further investigations are needed to clear up the correlation between synovial miR-27b and MMP-13 expression patterns.

Our results demonstrated that both serum and synovial fluid miR-27b go nearly in the same direction of expression, giving an idea that serum miR-27b could be used as a predictor for the joint state and a very early biomarker for OA.

On the other hand, miR-27b-3p was reported to be highly expressed in both samples collected from OA patients and rat models. Suppression of miR-27b-3p promotes the expression of the osteogenic differentiation markers while inhibiting the expression of the adipogenic differentiation markers, inflammatory factors, cellular senescence of bone marrow mesenchymal stem cells (BMSCs), and alleviating OA pain in rats by demethylation of KDM4B (Lysine demethylase 4B)^78^. These observations may explain our previous finding that the lameness score in donkeys has decreased with the progression of OA^1^.

Concerning the COL10A1 expression level in the current study, it increased in the 2nd and 3rd months, with the highest level at the 1st month after MIA injection, then downregulated at the 5th and 7th months.

Overexpression of miR-27b upregulated the mRNA expression of the hypertrophy marker COL10A1 and the levels of COL10A1 protein in human bone marrow mesenchymal stem cells^65^. miR-27b expression was found to be inversely correlated with chondrocyte hypertrophic differentiation in postnatal rat knee articular cartilage^79^.

In agreement, COLX was significantly higher with KL2, KL3, and KL4 osteoarthritic patients compared to patients with no radiographic sign of OA (KL0). Type X collagen is normally not expressed in human healthy articular cartilage, but its expression is detected at protein and mRNA levels in human OA cartilage^80^.

Also, Fukui et al*.* studied the regional and zonal differences in gene expression in OA cartilage and demonstrated that the overall expression of COL10A1 was elevated but that expression showed significant local variation. Notably, the expression of type COL10A1 was higher in the less degenerated joint region than in the more degenerated areas, and this explains our finding concerning the significant downregulation of COL10A1 at the late stages of OA^81^.

It is worth mentioning that, hypertrophy markers such as MMP-13 and type X collagen have been observed in several models of OA in different species, such as mice^82^ and rats^83^.

For instance, osteoarthritis involves the dysfunction of articular chondrocytes leading to cartilage degradation through chondrocyte maturation and MMP production^82^. During growth-plate chondrogenesis, the chondrocytes become hypertrophic with the expression of collagen type X, the collagen matrix is removed through the production of MMP-13, and finally, it undergoes apoptosis and is replaced by bone^84,85^.

Despite the limitation concerning the small number of animals used in our experiment, as well as the response of MIA-induced OA donkeys, which may differ from the naturally occurring OA ones, and based on our previous findings, we were able to track for the first-time changes in the expression of some biochemical, transcriptomic, and epigenetic factors in the serum and synovial fluid of Egyptian donkeys for the establishment of grading diagnostic biomarkers.

In conclusion, based on our previous classification for the stages of OA, biochemical markers (total GAGs and CS) showed their low efficiency in the early diagnosis or prognosis of OA since serum GAGs can not differentiate between the early and late stages of OA and synovial GAGs can not differentiate between the normal and late OA stages. CS in the serum cannot be used as an early diagnostic biomarker since it increases only at the late stage; also synovial CS cannot differentiate between early and late OA. Conversely, transcriptomic and epigenetic factors showed promising utility as very early noninvasive diagnostic biomarkers**.** Serum and synovial miR-146b showed a significant up-regulation at the very early stages of OA. To our knowledge, this is the first study investigating the expression level of miR-27b in serum during the different stages of OA, and we suppose that it would be a promising biomarker for the very early diagnosis of OA as it was up-regulated immediately 1-week post-MIA injection and nearly showed the same expression pattern as in the synovial fluid. Synovial COL10A1 showed its early, significant upregulation, representing an early biomarker for OA. All the above-mentioned findings, in combination with our previous grading for Equidae OA where the early stage of the disease is represented in the first month from MIA induction to OA, give the superiority to the serum samples to be selected as a noninvasive method for the early diagnosis of OA.

## Data Availability

All data generated or analyzed during this study are included in this published article.

## Abbreviations

OA: Osteoarthritis
MIA: Monoiodoacetate
GAGs: Glycosaminoglycans
CS: Chondroitin sulfate
SF: Synovial fluid
RA: Rheumatoid arthritis
MMP: Matrix metalloproteinases

## References

[1] Yassin AM, AbuBakr HO, Abdelgalil AI, Khattab MS, El-Behairy AM, Gouda EM (2020). COL2A1 and caspase-3 as promising biomarkers for osteoarthritis prognosis in an Equus asinus model. Biomolecules.

[2] Maumus M, Roussignol G, Toupet K, Penarier G, Bentz I, Teixeira S, Oustric D, Jung M, Lepage O, Steinberg R, Jorgensen C (2016). Utility of a mouse model of osteoarthritis to demonstrate cartilage protection by IFNγ-primed equine mesenchymal stem cells. Front. Immunol..

[3] Rousseau JC, Sornay-Rendu E, Borel O, Chapurlat R (2017). Association of circulating micrornas with osteoarthritis. Osteoarthr. Cartil..

[4] Rice SJ, Beier F, Young DA, Loughlin J (2020). Interplay between genetics and epigenetics in osteoarthritis. Nat. Rev. Rheumatol..

[5] Van Meurs JB (2017). Osteoarthritis year in review 2016: Genetics, genomics, and epigenetics. Osteoarthr. Cartil..

[6] Pitsillides AA, Beier F (2011). Cartilage biology in osteoarthritis—lessons from developmental biology. Nat. Rev. Rheumatol..

[7] Lotz MK, Carames B (2011). Autophagy and cartilage homeostasis mechanisms in joint health, aging, and OA. Nat. Rev. Rheumatol..

[8] Nugent M (2016). MicroRNAs: Exploring new horizons in osteoarthritis. Osteoarthr. Cartil..

[9] Hu G, Zhao X, Wang C, Geng Y, Zhao J, Xu J, Zuo B, Zhao C, Wang C, Zhang X (2017). MicroRNA-145 attenuates TNF-α-driven cartilage matrix degradation in osteoarthritis via direct suppression of MKK4. Cell Death Dis..

[10] O'Brien J, Hayder H, Zayed Y, Peng C (2018). Overview of microRNA biogenesis, mechanisms of actions, and circulation. Front. Endocrinol..

[11] Zhao C, Sun X, Li L (2019). Biogenesis and function of extracellular miRNAs. ExRNA..

[12] Yu XM, Meng HY, Yuan XL, Wang Y, Guo QY, Peng J, Wang AY, Lu SB (2015). MicroRNAs’ involvement in osteoarthritis and the prospects for treatments. Evid.-Based Complement. Altern. Med..

[13] Endisha H, Rockel J, Jurisica I, Kapoor M (2018). The complex landscape of microRNAs in articular cartilage: Biology, pathology, and therapeutic targets. JCI Insight..

[14] Peffers MJ, Balaskas P, Smagul A (2018). Osteoarthritis year in review 2017: Genetics and epigenetics. Osteoarthr. Cartil..

[15] Dai L, Zhang X, Hu X, Zhou C, Ao Y (2012). Silencing of microRNA-101 prevents IL-1β-induced extracellular matrix degradation in chondrocytes. Arthritis Res. Ther..

[16] Wang Q, Wang W, Zhang F, Deng Y, Long Z (2017). NEAT1/miR-181c regulates osteopontin (OPN)-mediated synoviocyte proliferation in osteoarthritis. J. Cell. Biochem..

[17] Steck E, Boeuf S, Gabler J, Werth N, Schnatzer P, Diederichs S, Richter W (2012). Regulation of H19 and its encoded microRNA-675 in osteoarthritis and under anabolic and catabolic in vitro conditions. J. Mol. Med..

[18] Kang Y, Song J, Kim D, Ahn C, Park S, Chun CH, Jin EJ (2016). PCGEM1 stimulates proliferation of osteoarthritic synoviocytes by acting as a sponge for miR-770. J. Orthop. Res..

[19] Miyaki S, Sato T, Inoue A, Otsuki S, Ito Y, Yokoyama S, Kato Y, Takemoto F, Nakasa T, Yamashita S, Takada S (2010). MicroRNA-140 plays dual roles in both cartilage development and homeostasis. Genes Dev..

[20] Yang CR, Shih KS, Liou JP, Wu YW, Hsieh IN, Lee HY, Lin TC, Wang JH (2014). Denbinobin upregulates miR-146a expression and attenuates IL-1β-induced upregulation of ICAM-1 and VCAM-1 expressions in osteoarthritis fibroblast-like synoviocytes. J. Mol. Med..

[21] Tardif G, Hum D, Pelletier JP, Duval N, Martel-Pelletier J (2009). Regulation of the IGFBP-5 and MMP-13 genes by the microRNAs miR-140 and miR-27a in human osteoarthritic chondrocytes. BMC Musculoskelet. Disord..

[22] Yang F, Hu A, Zhao D, Guo L, Yang L, Wang B, Tian F, Liu B, Huang S, Xie H (2015). An insertion/deletion polymorphism at the microRNA-122 binding site in the interleukin-1α 3′-untranslated region is associated with a risk for osteoarthritis. Mol. Med. Rep..

[23] Li ZC, Han N, Li X, Li G, Liu YZ, Sun GX, Wang Y, Chen GT, Li GF (2015). Decreased expression of microRNA-130a correlates with TNF-α in the development of osteoarthritis. Int. J. Clin. Exp. Pathol..

[24] Lu X, Lin J, Jin J, Qian W, Weng X (2016). Hsa-miR-15a exerts protective effects against osteoarthritis by targeting aggrecanase-2 (ADAMTS5) in human chondrocytes. Int. J. Mol. Med..

[25] Park SJ, Cheon EJ, Lee MH, Kim HA (2013). MicroRNA-127-5p regulates matrix metalloproteinase 13 expression and interleukin-1β–induced catabolic effects in human chondrocytes. Arthritis Rheum..

[26] Moldovan L, Batte KE, Trgovcich J, Wisler J, Marsh CB, Piper M (2014). Methodological challenges in utilizing mi RNA s as circulating biomarkers. J. Cell Mol. Med..

[27] Buschmann D, Haberberger A, Kirchner B, Spornraft M, Riedmaier I, Schelling G, Pfaffl MW (2016). Toward reliable biomarker signatures in the age of liquid biopsies-how to standardize the small RNA-Seq workflow. Nucleic Acids Res..

[28] Murata K, Yoshitomi H, Tanida S, Ishikawa M, Nishitani K, Ito H, Nakamura T (2010). Plasma and synovial fluid microRNAs as potential biomarkers of rheumatoid arthritis and osteoarthritis. Arthritis Res. Ther..

[29] Li YH, Tavallaee G, Tokar T, Nakamura A, Sundararajan K, Weston A, Sharma A, Mahomed NN, Gandhi R, Jurisica I, Kapoor M (2016). Identification of synovial fluid microRNA signature in knee osteoarthritis: Differentiating early-and late-stage knee osteoarthritis. Osteoarthr.Cartil..

[30] Ntoumou E, Tzetis M, Braoudaki M, Lambrou G, Poulou M, Malizos K, Stefanou N, Anastasopoulou L, Tsezou A (2017). Serum microRNA array analysis identifies miR-140-3p, miR-33b-3p, and miR-671-3p as potential osteoarthritis biomarkers involved in metabolic processes. Clin. Epigenet..

[31] Chen C, Chen H (2019). Clinical diagnosis value of mir-29b-3p in peripheral blood mononuclear cells and synovial fluid among osteoarthritis patients. Clin. Lab..

[32] Zhang M, Liu L, Xiao T, Guo W (2012). Detection of the expression level of miR-140 using realtime fluorescent quantitative PCR in knee synovial fluid of osteoarthritis patients. Zhong nan da xue xue bao. Yi xue ban = J. Cent. South Univ. Med. Sci..

[33] Kim MC, Lee SW, Ryu DY, Cui FJ, Bhak J, Kim Y (2014). Identification and characterization of microRNAs in normal equine tissues by next generation sequencing. PLoS ONE.

[34] Pacholewska A, Mach N, Mata X, Vaiman A, Schibler L, Barrey E, Gerber V (2016). Novel equine tissue miRNAs and breed-related miRNA expressed in serum. BMC Genomics.

[35] Barrey E, Bonnamy B, Barrey EJ, Mata X, Chaffaux S, Guerin G (2010). Muscular microRNA expressions in healthy and myopathic horses suffering from polysaccharide storage myopathy or recurrent exertional rhabdomyolysis. Equine Vet. J..

[36] Desjardin C, Vaiman A, Mata X, Legendre R, Laubier J, Kennedy SP, Laloe D, Barrey E, Jacques C, Cribiu EP, Schibler L (2014). Next-generation sequencing identifies equine cartilage and subchondral bone miRNAs and suggests their involvement in osteochondrosis physiopathology. BMC Genomics.

[37] da Costa SH, Hess T, Bruemmer J, Splan R (2018). Possible role of MicroRNA in equine insulin resistance: A pilot study. J. Equine Vet..

[38] Castanheira C, Balaskas P, Falls C, Ashraf-Kharaz Y, Clegg P, Burke K, Fang Y, Dyer P, Welting TJ, Peffers MJ (2021). Equine synovial fluid small non-coding RNA signatures in early osteoarthritis. BMC Vet. Res..

[39] Chou CK, Chi SY, Huang CH, Chou FF, Huang CC, Liu RT, Kang HY (2016). IRAK1, a target of miR-146b, reduces cell aggressiveness of human papillary thyroid carcinoma. J. Clin. Endocrinol. Metab..

[40] Li Y, Zhang H, Dong Y, Fan Y, Li Y, Zhao C (2017). MiR-146b-5p functions as a suppressor miRNA and prognosis predictor in non-small cell lung cancer. J. Cancer.

[41] Liu X, Liu L, Zhang H, Shao Y, Chen Z, Feng X, Fang H, Zhao C, Pan J, Zhang H, Zeng C (2019). MiR-146b accelerates osteoarthritis progression by targeting alpha-2-macroglobulin. Aging.

[42] Akhtar N, Rasheed Z, Ramamurthy S, Anbazhagan AN, Voss FR, Haqqi TM (2010). MicroRNA-27b regulates the expression of matrix metalloproteinase 13 in human osteoarthritis chondrocytes. Arthritis Rheum..

[43] Chu CR, Williams AA, Coyle CH, Bowers ME (2012). Early diagnosis to enable early treatment of pre-osteoarthritis. Arthritis Res. Ther..

[44] Gässler N, Reissner C, Janzen N, Kähnert H, Kleesiek K (1993). A high performance liquid chromatography method for the determination of glycosaminoglycans in human blood. Clin. Chem. Lab. Med..

[45] Livak KJ, Schmittgen TD (2001). Analysis of relative gene expression data using real-time quantitative PCR and the 2^−^^ΔΔ^^CT^ method. Methods.

[46] Reijman M, Hazes JM, Bierma-Zeinstra SM, Koes BW, Christgau S, Christiansen C, Uitterlinden AG, Pols HA (2004). A new marker for osteoarthritis: Cross-sectional and longitudinal approach. Arthritis Rheum..

[47] Catterall JB, Stabler TV, Flannery CR, Kraus VB (2010). Changes in serum and synovial fluid biomarkers after acute injury (NCT00332254). Arthritis Res. Ther..

[48] Smedsrød B, Kjellen L, Pertoft H (1985). Endocytosis and degradation of chondroitin sulphate by liver endothelial cells. Biochem. J..

[49] Alwan WH, Carter SD, Bennett D, Edwards GB (1991). Glycosaminoglycans in horses with osteoarthritis. Equine Vet. J..

[50] Wluka AE, Stuckey S, Snaddon J, Cicuttini FM (2002). The determinants of change in tibial cartilage volume in osteoarthritic knees. Arthritis Rheum..

[51] Kulkarni P, Deshpande S, Koppikar S, Patil S, Ingale D, Harsulkar A (2016). Glycosaminoglycan measured from synovial fluid serves as a useful indicator for progression of osteoarthritis and complements Kellgren-Lawrence Score. BBA Clin..

[52] Uesaka S, Nakayama Y, Shirai Y, Yoshihara K (2001). Serum and synovial fluid levels of chondroitin sulfate in patients with osteoarthritis of the knee joint. J. Nippon Med. Sch..

[53] Baccarin RY, Rasera L, Machado TS, Michelacci YM (2014). Relevance of synovial fluid chondroitin sulphate as a biomarker to monitor polo pony joints. Can. J. Vet. Res..

[54] Tavallaee G, Rockel JS, Lively S, Kapoor M (2020). MicroRNAs in synovial pathology associated with osteoarthritis. Front. Med..

[55] Taganov KD, Boldin MP, Chang KJ, Baltimore D (2006). NF-κB-dependent induction of microRNA miR-146, an inhibitor targeted to signaling proteins of innate immune responses. Proc. Natl. Acad. Sci..

[56] Budd E, de Andrés MC, Sanchez-Elsner T, Oreffo RO (2017). MiR-146b is down-regulated during the chondrogenic differentiation of human bone marrow derived skeletal stem cells and up-regulated in osteoarthritis. Sci. Rep..

[57] Okuhara A, Nakasa T, Shibuya H, Niimoto T, Adachi N, Deie M, Ochi M (2012). Changes in microRNA expression in peripheral mononuclear cells according to the progression of osteoarthritis. Mod. Rheumatol..

[58] Zhang X, Wang C, Zhao J, Xu J, Geng Y, Dai L, Huang Y, Fu SC, Dai K, Zhang X (2017). miR-146a facilitates osteoarthritis by regulating cartilage homeostasis via targeting Camk2d and Ppp3r2. Cell Death Dis..

[59] Guan YJ, Li J, Yang XU, Du S, Ding J, Gao Y, Zhang Y, Yang K, Chen Q (2018). Evidence that miR-146a attenuates aging-and trauma-induced osteoarthritis by inhibiting Notch 1, IL-6, and IL-1 mediated catabolism. Aging Cell.

[60] Armstrong AP, Tometsko ME, Glaccum M, Sutherland CL, Cosman D, Dougall WC (2002). A RANK/TRAF6-dependent signal transduction pathway is essential for osteoclast cytoskeletal organization and resorptive function. J. Biol. Chem..

[61] Jiang J, Zhang J, Wu C, Chen C, Bao G, Xu G, Xue P, Zhou Y, Sun Y, Cui Z (2021). Knockdown of TRAF6 inhibits chondrocytes apoptosis and inflammation by suppressing the NF-κB pathway in lumbar facet joint osteoarthritis. Mol. Cell. Biochem..

[62] Zhu LJ, Dai L, Zheng DH, Mo YQ, Ou-Yang X, Wei XN, Shen J, Zhang BY (2012). Upregulation of tumor necrosis factor receptor-associated factor 6 correlated with synovitis severity in rheumatoid arthritis. Arthritis Res. Ther..

[63] Li X, Gibson G, Kim JS, Kroin J, Xu S, Van Wijnen AJ, Im HJ (2011). MicroRNA-146a is linked to pain-related pathophysiology of osteoarthritis. Gene.

[64] West C, McDermott MF (2017). Biosci. Rep..

[65] Lv F, Huang Y, Lv W, Yang L, Li F, Fan J, Sun J (2017). MicroRNA-146a ameliorates inflammation via TRAF6/NF-κB pathway in intervertebral disc cells. Med. Sci. Monit. Int. Med. J. Exp. Clin. Res..

[66] Miyaki S, Asahara H (2012). Macro view of microRNA function in osteoarthritis. Nat. Rev. Rheumatol..

[67] Li X, Kroin JS, Kc R, Gibson G, Chen D, Corbett GT, Pahan K, Fayyaz S, Kim JS, Van Wijnen AJ, Suh J (2013). Altered spinal microRNA-146a and the microRNA-183 cluster contribute to osteoarthritic pain in knee joints. J. Bone Miner. Res..

[68] Wang JH, Shih KS, Wu YW, Wang AW, Yang CR (2013). Histone deacetylase inhibitors increase microRNA-146a expression and enhance negative regulation of interleukin-1β signaling in osteoarthritis fibroblast-like synoviocytes. Osteoarthr. Cartil..

[69] Zhang F, Wang J, Chu J, Yang C, Xiao H, Zhao C, Sun Z, Gao X, Chen G, Han Z, Zou W (2015). MicroRNA-146a induced by hypoxia promotes chondrocyte autophagy through Bcl-2. Cell. Physiol. Biochem..

[70] Li J, Huang J, Dai L, Yu D, Chen Q, Zhang X, Dai K (2012). miR-146a, an IL-1β responsive miRNA, induces vascular endothelial growth factor and chondrocyte apoptosis by targeting Smad4. Arthritis Res. Ther..

[71] Jin L, Zhao J, Jing W, Yan S, Wang X, Xiao C, Ma B (2014). Role of miR-146a in human chondrocyte apoptosis in response to mechanical pressure injury in vitro. Int. J. Mol. Med..

[72] Pillai RS (2005). MicroRNA function: Multiple mechanisms for a tiny RNA?. RNA.

[73] Toegel S, Wu SQ, Otero M, Goldring MB, Leelapornpisid P, Chiari C, Kolb A, Unger FM, Windhager R, Viernstein H (2012). Caesalpinia sappan extract inhibits IL1β-mediated overexpression of matrix metalloproteinases in human chondrocytes. Genes Nutr..

[74] Zhou Y, Li S, Chen P, Yang B, Yang J, Liu R, Li J, Xia D (2019). MicroRNA-27b-3p inhibits apoptosis of chondrocyte in rheumatoid arthritis by targeting HIPK2. Artif. Cells Nanomed. Biotechnol..

[75] Song J, Lee M, Kim D, Han J, Chun CH, Jin EJ (2013). MicroRNA-181b regulates articular chondrocytes differentiation and cartilage integrity. Biochem. Biophys. Res. Commun..

[76] Kostopoulou F, Malizos KN, Papathanasiou I, Tsezou A (2015). MicroRNA-33a regulates cholesterol synthesis and cholesterol efflux-related genes in osteoarthritic chondrocytes. Arthritis Res. Ther..

[77] Martinez-Sanchez A, Dudek KA, Murphy CL (2012). Regulation of human chondrocyte function through direct inhibition of cartilage master regulator SOX9 by microRNA-145 (miRNA-145). J. Biol. Chem..

[78] Zhang G, Zhou Y, Su M, Yang X, Zeng B (2020). Inhibition of microRNA-27b-3p relieves osteoarthritis pain via regulation of KDM4B-dependent DLX5. BioFactors.

[79] Xu J, Lv S, Hou Y, Xu K, Sun D, Zheng Y, Zhang Z, Li X, Li Y, Chi G (2018). Biosci. Rep..

[80] He Y, Siebuhr AS, Brandt-Hansen NU, Wang J, Su D, Zheng Q, Simonsen O, Petersen KK, Arendt-Nielsen L, Eskehave T, Hoeck HC (2014). Type X collagen levels are elevated in serum from human osteoarthritis patients and associated with biomarkers of cartilage degradation and inflammation. BMC Musculoskelet. Disord..

[81] Fukui N, Ikeda Y, Ohnuki T, Tanaka N, Hikita A, Mitomi H, Mori T, Juji T, Katsuragawa Y, Yamamoto S, Sawabe M (2008). Regional differences in chondrocyte metabolism in osteoarthritis: A detailed analysis by laser capture microdissection. Arthritis Rheum..

[82] Kamekura S, Kawasaki Y, Hoshi K, Shimoaka T, Chikuda H, Maruyama Z, Komori T, Sato S, Takeda S, Karsenty G, Nakamura K (2006). Contribution of runt-related transcription factor 2 to the pathogenesis of osteoarthritis in mice after induction of knee joint instability. Arthritis Rheum..

[83] Appleton CT, McErlain DD, Pitelka V, Schwartz N, Bernier SM, Henry JL, Holdsworth DW, Beier F (2007). Forced mobilization accelerates pathogenesis: Characterization of a preclinical surgical model of osteoarthritis. Arthritis Res. Ther..

[84] Inada M, Wang Y, Byrne MH, Rahman MU, Miyaura C, López-Otín C, Krane SM (2004). Critical roles for collagenase-3 (Mmp13) in development of growth plate cartilage and in endochondral ossification. Proc. Natl. Acad. Sci..

[85] Wang Y, Middleton F, Horton JA, Reichel L, Farnum CE, Damron TA (2004). Microarray analysis of proliferative and hypertrophic growth plate zones identifies differentiation markers and signal pathways. Bone.

